# The landscape of human tissue and cell type specific expression and co-regulation of senescence genes

**DOI:** 10.1186/s13024-021-00507-7

**Published:** 2022-01-09

**Authors:** Peng Xu, Minghui Wang, Won-min Song, Qian Wang, Guo-Cheng Yuan, Peter H. Sudmant, Habil Zare, Zhidong Tu, Miranda E. Orr, Bin Zhang

**Affiliations:** 1 Department of Genetics and Genomic Sciences, Icahn School of Medicine at Mount Sinai, One Gustave L. Levy Place, New York, NY 10029 USA; 2 Mount Sinai Center for Transformative Disease Modeling, Icahn School of Medicine at Mount Sinai, One Gustave L. Levy Place, New York, NY 10029 USA; 3 Icahn Institute for Data Science and Genomic Technology, Icahn School of Medicine at Mount Sinai, One Gustave L. Levy Place, New York, NY 10029 USA; 4 Institute for Precision Medicine, Icahn School of Medicine at Mount Sinai, One Gustave L. Levy Place, New York, NY 10029 USA; 5 Department of Integrative Biology, University of California Berkeley, Berkeley, CA 94720 USA; 6 Center for Computational Biology, University of California Berkeley, Berkeley, CA 94720 USA; 7 Department of Cell Systems & Anatomy, The University of Texas Health Science Center, San Antonio, TX 78229 USA; 8 Glenn Biggs Institute for Alzheimer’s and Neurodegenerative Diseases, University of Texas Health Sciences Center, San Antonio, TX 78229 USA; 9 Section of Gerontology and Geriatric Medicine, Department of Internal Medicine, Wake Forest School of Medicine, Winston-Salem, NC 27157 USA; 10 Sticht Center for Healthy Aging and Alzheimer’s Prevention, Wake Forest School of Medicine, Winston-Salem, NC 27157 USA; 11 Salisbury VA Medical Center, Salisbury, NC 28144 USA; 12 Department of Department of Pharmacological Sciences, Icahn School of Medicine at Mount Sinai, One Gustave L. Levy Place, New York, NY 10029 USA

**Keywords:** Senescence, Network, Transcriptome, Human tissues, RNA-seq, Single cell

## Abstract

**Background:**

Cellular senescence is a complex stress response that impacts cellular function and organismal health. Multiple developmental and environmental factors, such as intrinsic cellular cues, radiation, oxidative stress, oncogenes, and protein accumulation, activate genes and pathways that can lead to senescence. Enormous efforts have been made to identify and characterize senescence genes (SnGs) in stress and disease systems. However, the prevalence of senescent cells in healthy human tissues and the global SnG expression signature in different cell types are poorly understood.

**Methods:**

This study performed an integrative gene network analysis of bulk and single-cell RNA-seq data in non-diseased human tissues to investigate SnG co-expression signatures and their cell-type specificity.

**Results:**

Through a comprehensive transcriptomic network analysis of 50 human tissues in the Genotype-Tissue Expression Project (GTEx) cohort, we identified SnG-enriched gene modules, characterized SnG co-expression patterns, and constructed aggregated SnG networks across primary tissues of the human body. Our network approaches identified 51 SnGs highly conserved across the human tissues, including *CDKN1A* (*p21*)-centered regulators that control cell cycle progression and the senescence-associated secretory phenotype (SASP). The SnG-enriched modules showed remarkable cell-type specificity, especially in fibroblasts, endothelial cells, and immune cells. Further analyses of single-cell RNA-seq and spatial transcriptomic data independently validated the cell-type specific SnG signatures predicted by the network analysis.

**Conclusions:**

This study systematically revealed the co-regulated organizations and cell type specificity of SnGs in major human tissues, which can serve as a blueprint for future studies to map senescent cells and their cellular interactions in human tissues.

**Supplementary Information:**

The online version contains supplementary material available at 10.1186/s13024-021-00507-7.

## Background

Cellular senescence is a complex stress response associated with four inter-dependent hallmarks: cell-cycle withdrawal, macromolecular damage, secretory phenotype, and deregulated metabolism [1]. The first report of cellular senescence described human fibroblasts reaching the end of their replicative lifespan in vitro [2]. Subsequent studies demonstrated that cells could prematurely enter senescence when exposed to adverse stimuli, including radiation, oxidative stress, telomere attrition, and oncogene signaling [3]. Senescence contributes to physiological processes such as embryonic development, regeneration, cell fate reprogramming [4], and is beneficial in wound healing and tumor suppression [5, 6]. Meanwhile, the accumulation of senescent cells may lead to various human diseases, including pulmonary fibrosis, hepatic steatosis, diabetes, and neurodegenerative diseases [7–10]. As senescence is closely associated with human diseases from multiple organs, the removal of senescent cells has been proposed to improve senescence-associated pathologies [11, 12].

Cellular senescence can be activated and maintained through diverse pathways [13]. Among them, p53/*CDKN1A* (p21) and *CDKN2A* (p16) /pRB pathways are central for cell cycle arrest. During the process of cellular senescence, senescent cells often display enlarged and flattened cell shape, vacuole accumulation, enhanced beta-galactosidase activity at pH 6.0 (SAβ-gal), and senescence-associated heterochromatin foci [14]. They also secrete a cocktail of inflammatory and stromal regulators [15], referred to as the senescence-associated secretory phenotype (SASP) [16]. These SASP factors, including interleukins, chemokines, growth factors, and proteases, are mainly regulated by NF-κB, C/EBPβ (CEBPB), p38^MAPK^, and mTOR signaling pathways [17–21]. Although senescent cells exhibit morphological changes with activation of cell cycle inhibitors and relevant SASP, no single marker is reliable for the in vivo recognition of senescent cells [22]. The knowledge of how senescence genes are expressed and co-regulated in various human tissues is limited.

The recent advances in sequencing technologies make genome-wide profiling of senescence-related signatures feasible. The transcriptomic and proteomic patterns of senescent cells have been studied in multiple model systems under different conditions [23–25]. The senescence gene (SnG) database CellAge was constructed by a comprehensive literature search of gene manipulation experiments [26]; most genomic profiling and genetic manipulations were based on in vitro systems such as cell lines. The in vivo expression patterns of SnGs across multiple human tissues remain unknown, and the cell-type specific molecular signatures of cellular senescence have not been revealed. In this study, we applied our previously established system biology approach [27–30] to identify co-expressed networks of SnGs in 50 non-diseased human tissues. We identified co-expression structures of SnGs and constructed a consensus senescence network conserved across multiple healthy tissues. To characterize the cell-type specific signatures of cellular senescence, we further analyzed single-cell RNA-seq (scRNA-seq) in six human tissues and performed an integrative analysis with tissue-specific co-expressed gene modules.

## Materials and methods

### GTEx bulk RNA-seq processing

To analyze bulk RNA-seq datasets of human tissues, we downloaded the raw count of RNA-seq data from the GTEx (v8) database. After filtering the tissues with less than 20 samples, we obtained RNA-seq samples from 50 tissues. Each tissue contained 20 to 663 samples. The samples were collected from non-diseased tissues of 948 individuals 20–80 years old. For each tissue, we used genes with an expression level higher than one log2CPM (count per million) in more than 25% of the samples for analysis. The read count data were normalized using a trimmed mean of M-values normalization (TMM) method to adjust for sequencing library size difference [31]. The normalized gene expressions were log2 transformed. We applied a linear model to adjust for the covariates including “SMCENTER” (collection sites), “SMRIN” (RNA integrity), “SMTSISCH” (ischemic time), “SMEXNCRT” (exonic rate), “SMRRNART” (rRNA rate), “SMNTERRT” (intergenic rate) and “SEX” (gender). The residuals from the regression model were used for downstream analyses, including network construction and age correlation. As the age of GTEx donors was recorded in a ten-year range, the mean value of the age range was used for correlation analysis.

### MEGENA co-expression network analysis

We constructed gene co-expression networks from normalized and covariate-adjusted gene expression data. As age may influence senescence geneexpression, we constructed two sets of gene co-expression networks through the established multiscale gene co-expression network analysis (MEGENA) [27–30], one based on the data adjusted for age and the other from the data without age adjustment. Briefly, Pearson correlation coefficients (PCCs) were computed for all gene pairs. Then significant PCCs were filtered by a false discovery rate (FDR) cutoff of 0.05. The ranked significant PCCs were iteratively tested for planarity to grow a Planar Filtered Network (PFN) using the PMFG algorithm [32]. The resulted PFN was analyzed by Multiscale Clustering Analysis (MCA) to identify co-expression modules at different scales of compactness. The Multiscale Hub Analysis (MHA) was performed to identify hub genes highly connected in each cluster. As our goal was to construct co-expression networks for the whole senescence landscape, we implemented the MEGENA pipeline by constructing “unsigned” networks (default parameter) where the connections of nodes only represent their relatedness, regardless of positive or negative correlations.

### Module annotation and enrichment analysis

To annotate the biological functions of the co-expressed modules, we applied ClusterProfiler to perform Fisher’s exact test of the module genes against the REACTOME pathways from the Molecular Signatures Database (MSigDB) [33]. To identify cell-type enrichment of network modules, we obtained marker genes of different cell types from the PanglaoDB database [34], and performed Fisher’s exact test by ClusterProfiler to compare the module genes and marker genes in each cell type. To analyze the module correlation with age, we calculated the module eigengene (the first principal component of module gene expression profile) by Principle Component Analysis (PCA). Then we performed Spearman correlation analysis to calculate the correlation between module eigengene and the donor age. Multi-testing *p*-values were adjusted by the BH method.

### Clustering of the CellAge SnGs and SnG-enriched modules

To study the distribution patterns of the CellAge SnGs, we performed a two-way clustering analysis of the CellAge SnGs and their enriched modules. We first constructed a binary presence matrix that contained the CellAge SnGs as the rows and the SnG-enriched modules as the columns. In the presence matrix, 1 indicated the presence of SnGs in each module, while 0 indicated the absence. Then the matrix was used for the euclidean distance based k-means (k = 4) clustering analysis implemented by the ComplexHeatmap [35] (v2.6.2) package in R. The clustering analysis was performed to identify the clusters of the CellAge SnGs and SnG-enriched modules, respectively. The k-means clustering was performed 100 times to generate a final consensus cluster. To visualize the dendrogram, the ComplexHeatmap showed the hierarchical clustering for the heatmap and then split the dendrogram based on the k-means clusters.

### Network aggregation and gene neighborhood analysis

To identify the conserved co-expression networks in multiple tissues, we performed the network aggregation based on the MEGENA co-expression networks and the 125 SnG-enriched modules. For each tissue, we identified the genes from the SnG-enriched modules. Then we constructed the co-expression network among these genes based on the links of the MEGENA network. The resultant networks in different tissues were merged by calculating the conservation weights for the nodes and edges. The node conservation weight was defined as the total frequency of the node gene in the tissues showing SnG-enrichment. Similarly, the edge conservation weight was defined as the total frequency of the gene-gene co-expression link in different co-expression networks. To obtain a global conserved co-expression network, we filtered the less conserved nodes with weights < 5, and constructed the final aggregated network from all the SnG-enriched modules.

To generate consensus networks of gene neighborhoods of *CDKN1A* and *TP53*, we first extracted the neighbor genes of the two genes in each MEGENA network. For each MEGENA network, we extracted 3-layer neighbor genes by iteratively searching the network edges that were connected to the target genes. The network among the neighbor genes was constructed based on the MEGENA network links. Then we merged the neighborhood networks of *CDKN1A* and *TP53* in all the tissues and calculated the conservation weights for the network nodes and edges. The node conservation weight corresponded to the total frequency of the node being the neighborhood genes in all the tissues. To obtain a global conserved co-expression network, we filtered the less conserved nodes with weights < 5, and constructed the final consensus neighborhood network from all tissues.

### Single-cell RNA-seq analysis

We curated several scRNA-seq datasets of different human tissues from four different publications. For the brain tissue, the single-nuclei RNA-seq (snRNA-seq) dataset was collected from three adults aged 19, 36, and 64 years old [36]. The raw counts were downloaded from the website http://development.psychencode.org/. Gene expressions were normalized by the global-scaling normalization method “LogNormalize” in Seurat (v3.9.9) [37], which normalized the gene expressions for each cell by the total expression, followed by multiplying by a scale factor (10,000 by default) and log-transformation. The cell type annotation for the sequenced cells was directly retrieved from the previous publication [36]. To identify marker genes of each cell type, the “FindAllMarkers” function from Seurat was applied to identify differentially expressed genes using a Wilcoxon Rank Sum test. Only significantly upregulated genes (FDR < 0.05) with 0.25 log fold change and 0.25 minimum expression fraction were retained as marker genes.

To analyze scRNA-seq from testis tissue, we downloaded raw counts from GEO (https://www.ncbi.nlm.nih.gov/geo/) with accession number GSE112013. The analysis pipeline follows the Seurat recommendations. Briefly, gene expressions were normalized by “LogNormalize” method. The top 2,000 variable genes were identified by the “FindVariableFeatures” function. After scaling gene expressions, a linear dimensional reduction was performed using the variable genes by “RunPCA” functions, which generated 30 principle components. For single-cell clustering, the “FindNeighbors” function was used to construct a K-nearest neighbor (KNN) graph based on the euclidean distance with the top 10 principal components. Then the “FindClusters” function was applied to optimize modularity by the Louvain algorithm. The resolution parameter for the clustering granularity was set to 0.05. Finally, the UMAP method was used for non-linear dimensional reduction and cluster visualization. The cell types were determined by expressions of the marker genes from the previous publication [38]. The “FindAllMarkers” function was applied to identify marker genes using the Wilcoxon Rank Sum test. The criteria to filter marker genes were the same as the brain scRNA-seq pipeline.

To analyze scRNA-seq from pancreas tissue, we downloaded raw counts from GEO with accession number GSE84133. The analysis was similar to the testis pipeline, including gene expression normalization, linear dimensional reduction, KNN graph construction, cell clustering, and cell-type annotation. To annotate different cell clusters, we used the same marker genes as the previous publication [39]. For esophagus, lung, and spleen tissues, the analysis was similar to brain pipeline. Raw counts were directly downloaded from https://www.tissuestabilitycellatlas.org/. The cell types for each tissue were retrieved from the publication [40]. Then marker genes for each cell type were identified by the “FindAllMarkers” function using the Wilcoxon Rank Sum test.

### Gene coexistence analysis

We performed gene coexistence analysis to verify that the genes from the aggregated network were co-expressed with senescence markers in single cells. As dropout effects frequently influence gene expression in single cells, we defined the strength of gene co-expression as the coexistence proportion, which calculates the percentage of cells expressing two genes of interest (normalized expressions higher than zero) simultaneously. We quantified the coexistence proportion for each senescence marker gene and each gene in the aggregated network or 1,000 random background genes from the whole genome. Wilcoxon test was used to calculate the significance of altered coexistence proportions between aggregated network genes and background genes in different cell types.

### Cell communication analysis

We analyzed the cell communications based on the marker genes of each cell type. First, we downloaded the ligand-receptor interactions in humans from the CellChatDB database [41], which contains manually curated literature-supported ligand-receptor pairs. To assess whether two cell types had significant interactions, the marker genes from the source cell type were searched against the marker genes from the target cell type based on the ligand-receptor pairs in the CellChatDB. Meanwhile, 10,000 permutations were performed from the expressed genes to calculate the background frequency of ligand-receptor pairs. The significance of cell type communication was determined by the permutation test, which compared the observed frequency with the background signal.

### Spatial transcriptomic analysis

We downloaded the spatial transcriptome dataset of the human postmortem dorsolateral prefrontal cortex from http://research.libd.org/globus/, which profiled gene expressions on 10-μm serial tissue sections using 10x Genomics Visium platform [42]. Section slide 151,507 from a 35-year-old donor was used to investigate cell-type localizations. Gene expressions from each voxel were normalized by the sctransform [43] in Seurat, which uses regularized negative binomial models to account for technical artifacts while preserving biological variance. Then top 30 principal components were calculated and used to construct the KNN graph. The Louvain algorithm was used to cluster the voxels. To learn cell type compositions of each voxel, the anchor-based integration workflow from Seurat was used to calculate probabilistic annotations from the scRNA-seq reference. To achieve this, the scRNA-seq dataset [36] was processed by a similar pipeline, which normalized gene expression by the sctransform and clustered cells by the Louvain algorithm. Then the “FindTransferAnchors” function from Seurat was used to project the PCA structure of the scRNA-seq onto the spatial transcriptomic dataset. The “TransferData” function was used to classify the voxel cells based on the scRNA-seq cell type annotations.

To calculate the M82-enriched spatial voxels, we applied the GSEA method [44] similar to the previous publication [45]. Briefly, we generated the entire ranked list for each voxel by ranking the expressed genes according to their normalized and scaled expressions. Then, we used the M82 gene set to calculate the enrichment score by “fgsea” package [46] in the R program. The enrichment score reflected the degree to which the M82 gene set was overrepresented at the extremes (top or bottom) of the entire ranked list. Conceptually, the score was calculated by walking down the ranked list, increasing a running-sum statistic when encountering a gene in the M82, and otherwise decreasing it [44]. The enrichment score corresponded to a weighted Kolmogorov–Smirnov-like statistic, standing for the maximum deviation from zero encountered in the random walk. The statistical significance (nominal *p* value) of the enrichment score was estimated by an empirical phenotype-based permutation test procedure with 1,000 permutations.

## Results

### The co-expression networks of SnGs in 50 human tissues

**Fig. 1:**
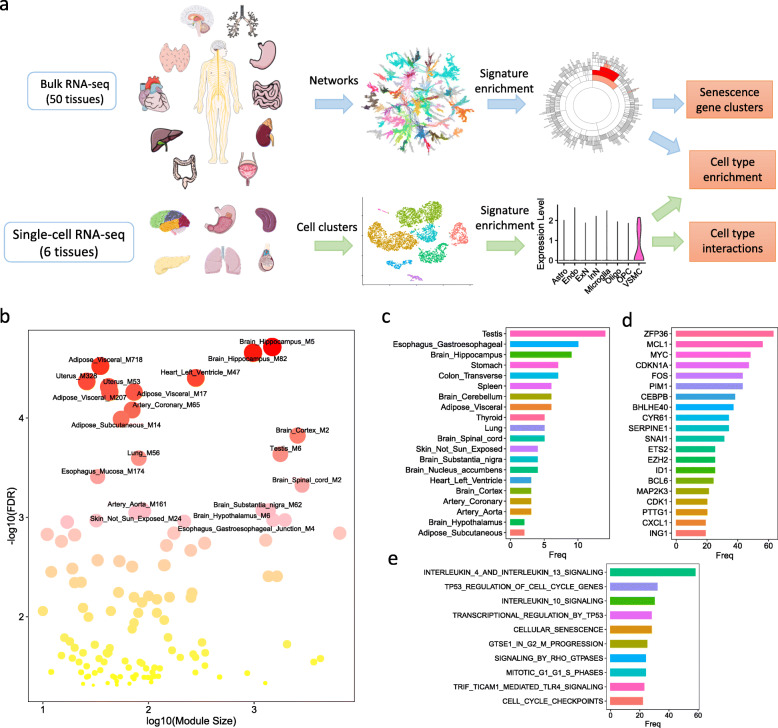
Gene co-expression network analysis of SnGs in human tissues. **a** An integrative network biology approach to study SnGs in human tissues. For bulk RNA-seq datasets, a gene co-expression network was constructed in each tissue to identify co-expressed gene modules enriched for the CellAge SnGs and marker genes of different cell types (subpopulations). For each scRNA-seq dataset, unsupervised clustering and differential gene expression analyses were performed to identify cell clusters (subpopulations) and their marker genes. Then the cell-type specific markers were tested for the enrichment in the SnG-enriched modules, revealing cell-type specific SnG signatures in each tissue. The bulk RNA-seq based network analysis and the scRNA-seq based cell type analysis were complementary and cross-validated, providing rich information on co-expression structures and cell-type specificity of SnGs. **b** Modules enriched for the CellAge SnGs in the 50 tissue-specific gene co-expression networks. Each dot indicates a SnG-enriched module, with the x-axis showing the module size and the y-axis showing the enrichment significance. False Discovery Rate (FDR) in the y-axis was calculated as multiple-testing corrected FET *p*-value. Color intensity and size of each dot are scaled with the enrichment p-value. The top 20 modules most significantly enriched for the CellAge SnGs are labeled. **c** The top 20 tissues with the gene modules enriched for the CellAge SnGs. The x-axis shows the number of modules enriched for the CellAge SnGs in each tissue. **d** The top 20 genes most frequently detected in the SnG-enriched modules. The x-axis indicates the number of enriched modules which contain a given gene. **e** The top 10 REACTOME pathways for the SnG-enriched modules. The x-axis shows the number of the SnG-enriched modules enriched for a given pathway

To investigate senescence gene signatures in various human tissues and cell types, we developed a network biology based framework to integrate bulk and scRNA-seq data (Fig. 1a). We performed a well-established Multiscale Embedded Gene Co-expression Network Analysis (MEGENA) [27–30] on 50 non-diseased human tissues using the bulk RNA-seq data in the GTEx database [47–49]. MEGENA identified 38,152 gene modules from 50 tissue-specific gene co-expression networks, with an average of 763 modules per tissue (network). The member genes of each module were then compared with the CellAge database [26], which contains 279 experimentally validated SnGs. We identified 125 network modules with significant enrichment (multiple-testing corrected Fisher’s exact test (FET) *p*-value < 0.05) of the CellAge SnGs (Fig. 1b, Table S1). These enriched modules were from 32 tissues. The gene modules were then rank-ordered by the significance of the enrichment for the CellAge SnGs. The top-ranked modules were from tissues such as the brain, the adipose, the uterus, the heart, the testis, the lung, and the esophagus, suggesting various origins of senescence signatures. The testis had the most modules enriched for the CellAge SnGs, followed by the esophagus, the hippocampus, the stomach, the colon, and the spleen (Fig. 1c). The CellAge SnGs were enriched in the modules across different brain regions (e.g., the hippocampus, the cortex, the spinal cord, the substantia nigra, and the hypothalamus). In the SnG-enriched modules, the most frequently detected genes included not only well-defined senescence markers (e.g., cell cycle regulator *CDKN1A* [50], SASP regulators *CEBPB* and *MAP2K3* [17, 51]), but also gene regulators from diverse senescence pathways (e.g., *SERPINE1* in replicative senescence [52], *ETS2* in oncogene stress [53], *CYR61*/*CCN1* in wound healing [54], *FOS* in enhancer organization [55], *ZFP36* in mRNA stability regulation [56], and *ING1* in epigenetic regulation [57]) (Fig. 1d). Consistent with the functional roles, the SnG-enriched modules were most related to biological pathways in interleukin signaling, TP53 regulations, cellular senescence, and cell cycle regulations (Fig. 1e). These results revealed the co-expression and co-regulated organizations of SnGs in human tissues.

Cellular senescence is triggered by diverse factors, including organismal aging [58]. Therefore, we calculated the correlation between age and network modules to evaluate the age influence on individual modules. We performed the Spearman correlation analysis of age and module eigengenes. We found that 34 (27%) of the 125 SnG-enriched modules were correlated with age (Spearman correlation *p* < 0.05; Table S1). The age-correlated modules were from 15 different tissues, including the lung (*n* = 5), the spleen (*n* = 4), the brain hippocampus (*n* = 3), the esophagus (*n* = 3), and the heart left ventricle (*n* = 3). For example, M5 was the module most significantly enriched for SnGs (*p* = 5.8e-08, Fisher’s exact test) in the hippocampus (Fig. 1b, Table S1). Its eigengene was also significantly correlated with age (*r* = 0.23, *p* = 1.2e-03, Spearman correlation), suggesting increased expression of M5 with advanced chronological age.

### The co-expression patterns of the CellAge SnGs

**Fig. 2:**
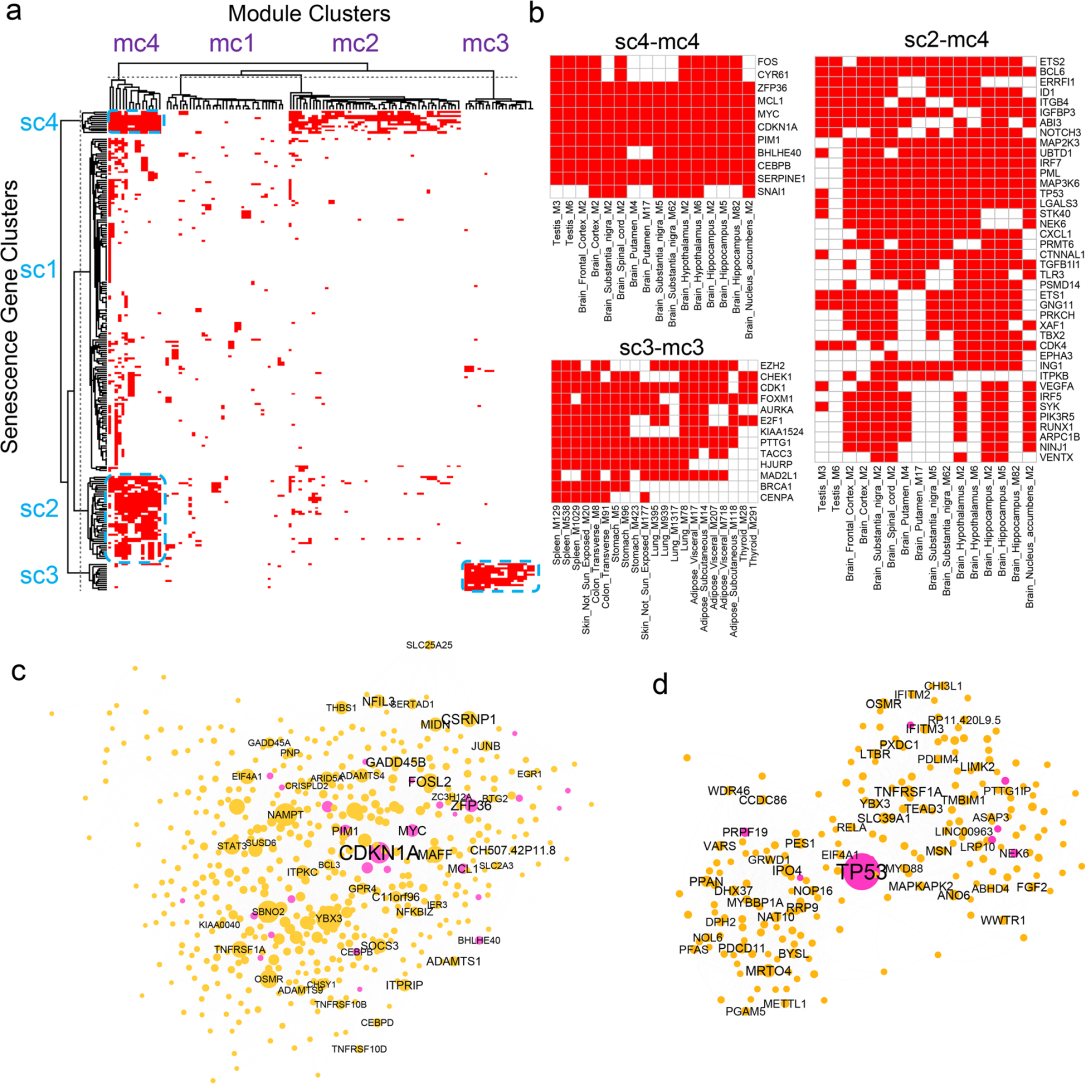
The co-expression patterns of the CellAge SnGs and SnG-enriched modules. **a** Heatmap plot of the clusters of the CellAge SnGs and SnG-enriched modules, with each row representing for a SnG and each column for a SnG-enriched module. The hierarchical dendrograms were shown for the heatmap and further split based on the k-means clusters (k = 4, 100 iterations). The red color of each pixel indicates the presence of a SnG in a module. The blue dotted rectangles indicate three representative gene-module clusters shown in panel 2b. **b** Heatmap plots of three representative gene-module clusters from sc4-mc4, sc3-mc3, and sc2-mc4, respectively. Each row represents a SnG, and each column represents a module in the selected cluster. **c-d** The consensus networks of *CDKN1A* (**c**) and *TP53* (**d**) neighborhood genes across 50 tissues. The network of each gene was constructed by aggregating 3-layer neighbor genes across 50 co-expression networks. The node weight was calculated based on the gene conservation in the 50 networks. The genes with node weight > = 5 were used for aggregation. The node size in the network plot is proportional to the node weight, and the gene symbols with the top 50 node weights are labeled. The pink node color indicates the CellAge SnGs

Although the CellAge SnGs were identified by various experimental approaches [26], how they are expressed and co-regulated in different human tissues is largely unknown. The network analysis identified 125 SnG-enriched gene modules in 32 human tissues. These SnG-enriched modules included 225 or 81% of the SnGs from the CellAge database. To explore the co-expression patterns of the 225 CellAge SnGs, we performed a two-way clustering analysis of the 225 SnGs and the 125 SnG-enriched modules using the k-means method. By calculating Euclidean distance from the presence matrix of SnGs in each module, the k-means method (k = 4, 100 iterations) identified four SnG clusters (namely, sc1–4) and four module clusters (namely, mc1–4) (Fig. 2a, Table S2–3). Among the four SnG clusters, the cluster sc4 contained 11 genes (e.g., *CDKN1A* and *CEBPB*) and was most widely present in the SnG-enriched modules in 30 different tissues (Fig. 2b). On average, each CellAge SnG in the cluster sc4 was detected in the SnG-enriched modules from 21 different tissues. Functional studies supported the central roles of the cluster sc4 in regulating various senescence pathways. For example, *CDKN1A* is a key senescence marker gene that represses cell cycle progression [50]; *ZFP36* regulates mRNA stability of cell cycle and cytokine genes [56]; *CYR61*/*CCN1* induces fibroblast senescence during wound healing [54]; *MCL1* is an apoptosis repressor from the BCL2 family [59]; *CEBPB* is a crucial regulator of senescence and SASP [17, 51]; *SERPINE1* regulates replicative senescence downstream of *TP53* [52]; *FOS* forms the AP-1 complex that drives the senescence enhancer landscape [55]. Intriguingly, the cluster sc4 also contained oncogenes *FOS*, *MYC,* and *SNAI1*, consistent with the close relationship between cellular senescence and cell proliferation.

Apart from the SnG cluster sc4, the cluster sc2 contained 40 CellAge SnGs and was also widely expressed in the SnG-enriched modules from 26 different tissues. On average, each SnG from sc2 was detected in the SnG-enriched modules from eight different tissues. Many important senescence regulators fell into cluster sc2 (Fig. 2b). For example, *TP53* is a senescence marker gene that represses cell cycle in response to cellular stress and DNA damage [60]; *IRF5* and *IRF7* encode interferon regulatory factors that inhibit cell growth and induce senescence [61]; *MAP2K3* (*MKK3*) can activate the stress-inducible p38^MAPK^ pathway for SASP induction [19, 62]; *RUNX1* (*AML1*) is an oncogene that induces a senescence-like growth arrest in fibroblasts [63]; *ING1a* can bind to histone deacetylases and induce senescent cell morphology [57]; *NOTCH3* from the NOTCH signaling pathway regulates *CDKN1A* expression in senescent cells [64]. Among the four SnG clusters, sc1 was the largest cluster with 161 CellAge SnGs. However, on average, the SnGs from sc1 only came from two different tissues, suggesting the expression of this cluster was not prevalent in the SnG-enriched modules. Cluster sc3 was well separated from the other clusters. Consistent with the distinct clustering position, sc3 contained 13 CellAge SnGs, including *BRCA1*, *FOXM1*, *E2F1*, and *CDK1*, which are mainly related to cell cycle progression and cellular senescence inhibition. The CellAge database contains inducer and inhibitor genes of cellular senescence, and both of them are crucial for regulating cellular senescence. Interestingly, the senescence inhibitors fromsc3 were preferentially co-expressed in a number of tissue-specific gene modules independent of other senescence-related genes, indicating their coordinated activities in regulating cellular senescence.

As the clusters sc4 (*n* = 11) and sc2 (*n* = 40) contained the CellAge SnGs in more than half of the surveyed tissues, we considered 51 genes from the two clusters as the conserved SnGs. Among them, *CDKN1A* from sc4 and *TP53* from sc2 are well-recognized marker genes for cellular senescence. Therefore, we explored the conservation of co-expression relationships of the two genes in different tissues. By extracting 3-layer neighborhood genes in the co-expression networks, we constructed consensus networks of *CDKN1A* and *TP53* in at least five tissues (Methods, Table S4–5). The consensus network of the *CDKN1A* neighborhood had 531 genes, including 28 CellAge SnGs (Fig. 2c). Interestingly, the *CDKN1A* neighborhood contained all the genes (*n* = 11) from the SnG cluster sc4 and 14 (35%) genes from the cluster sc2, suggesting a central role of *CDKN1A*. For example, *CDKN1A* was co-expressed with *MCL1*, *CEBPB*, *FOS*, and *SERPINE1* in 27, 21, 18, and 15 tissues, respectively. The top enriched pathways of the *CDKN1A* network neighborhood genes included the interleukin signaling, the Toll-like receptors, the metal ions response, and cellular senescence. The interferon signaling genes *STAT3*, *IL4R*, *IL1R1*, *CCL2*, and *IL6,* were co-expressed with *CDKN1A* in 21, 18, 16, 13, and 9 tissues, respectively. In the consensus network of *TP53*, 225 genes were identified as the network neighbors in more than four tissues, including nine CellAge SnGs. These neighborhood genes contained seven (17.5%) genes from the SnG cluster sc2, but none of them came from the cluster sc4. The consensus networks of *CDKN1A* and *TP53* shared 63 neighborhood genes, including several essential cytokine regulators (e.g., interleukin signaling regulator *STAT3*, NF-κB signaling regulator *RELA*, and TNF receptor gene *TNFRSF1A*).

### Aggregation of the SnG-enriched modules

**Fig. 3:**
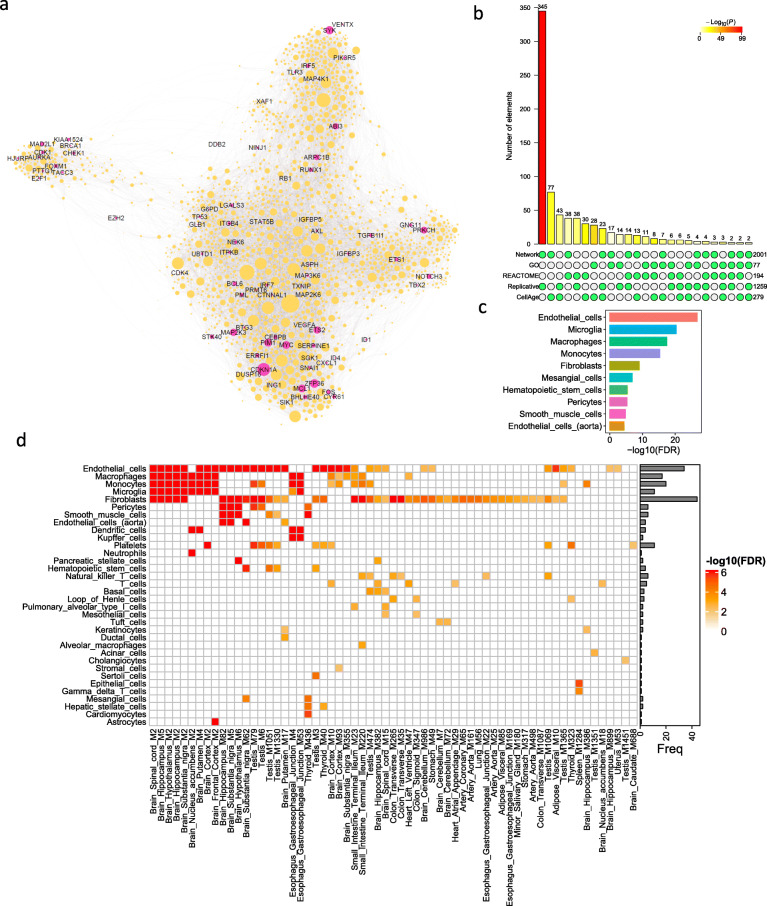
The aggregated SnG network and cell-type enrichment. **a** The aggregated network from 125 SnG-enriched modules. In the network plot, the node corresponds to a gene from SnG-enriched modules, and the link corresponds to the co-expression relationship between genes in MEGENA co-expression networks. The aggregated network was generated by merging co-expression links between SnG-enriched module genes conserved in at least five tissues (Methods). The node size in the network plot is proportional to the node conservation in different tissues, and the gene symbols of the CellAge SnGs are labeled. **b** Comparison of the aggregated network with four SnG annotation datasets. A bar chart shows possible intersections among the five annotation datasets (including the aggregated network) in a matrix layout, with the solid and empty circles indicating the presence and absence of the gene sets for each intersection, respectively. The numbers to the right of the matrix indicate set sizes. The color bars on the top of the matrix show the intersection sizes. The color intensity is proportional to the one-tailed hypergeometric *p*-value significance. **c** Barplot showing the top 10 cell types whose marker genes are significantly enriched (FDR < 0.01) for the aggregated network genes. The FDR was calculated as the adjusted FET *p*-value. **d** The heatmap plot showing the cell types whose marker genes are enriched (FDR < 0.01) in the SnG-enriched modules. Each row shows the cell types, and each column shows the network module. The colors are scaled with the enrichment value. The barplot on the right shows the total frequency of cell-type enrichment

As the SnG-enriched modules were identified by tissue-specific co-expression networks, we further merged these modules into a global network to reveal the landscape of SnG co-expression structure across multiple tissues. We first extracted conserved genes from the SnG-enriched modules in at least five tissues and then constructed an aggregated network by merging the co-expression links between these conserved genes in all the tissues (Methods). The aggregated network thus represented the global correlationship between highly conserved genes from the SnG-enriched modules. We identified 2,001 highly connected genes in the aggregated network (Fig. 3a, Table S6). All the genes (*n* = 11) from cluster sc4 and 38 (95%) genes from cluster sc2 were present in the aggregated network, suggesting conserved signatures of the two gene clusters. In contrast, cluster sc1 only had 16 (10%) genes in the aggregated network. Gene set enrichment analysis (GSEA) showed that genes from the clusters sc4 and sc2 were significantly enriched (*p* = 1.5e-9, 1,000 permutations) in the top-ranked nodes with high conservation weights. In the aggregated network, *ZFP36*, *CDKN1A*, *MCL1*, *FOS,* and *MYC* from the CellAge database were ranked among the top 10 conserved nodes. To further understand the aggregated network, we compared it with SnG annotations from the GO (*n* = 77), the REACTOME (*n* = 194), and the CellAge (*n* = 279), as well as a replicative senescence signature (*n* = 1259) from the recent meta-analysis [65]. The aggregated network significantly overlapped the senescence signatures from all annotation datasets (Fig. 3b). Remarkably, the aggregated network was most enriched for the replicative senescence signature (fold enrichment (FE) = 3.4, FET *p* = 2.3e-99). Meanwhile, the aggregated network contained 77 genes from the CellAge database (FE = 3.4, FET *p* = 2.0e-22), 38 genes from REACTOME database (FE = 2.4, FET *p* = 2.6e-7), and 17 genes (FE = 2.7, FET *p* = 1.1e-4) from GO database. These results suggested the aggregated network shares similarities with other SnG annotations and captures the replicative senescence in multiple tissues, which were mostly described by in vitro cell lines [65].

Among the 2,001 genes from the aggregated network, 488 genes are annotated as putative SASP factors (Gene Ontology Consortium and QuickGO database). About 21.1% (103) of these SASP factors were upregulated in the senescent human fetal lung (IMR-90) cells induced by γ-irradiation (Table S7) [66]. In contrast, only 10% of the total SASP factors (*n* = 3,513) in the human genome were upregulated, suggesting the aggregated network was significantly enriched for the SASP factors in senescent cells (FET *p* = 1.9e-15). Among the 103 upregulated SASP factors from the aggregated network, only 5 (5%) genes were annotated by CellAge experiments, and the majority have not been experimentally validated. More than half (59) of the upregulated SASP factors depend on *CDKN1A* expression (Table S7) [66], suggesting a major regulatory role of *CDKN1A* in SnG co-expressions.

Based on the aggregated SnG network, we explored the cell types that contribute to the co-expression of the network genes. By comparing cell-type marker genes from the PanglaoDB database [34], we identified 14 cell types associated with the genes in the aggregated network (adjusted FET *p*-value (aFETp) < 0.01). The top five cell types were endothelial cells (aFETp = 1.9e-27), microglia (aFETp = 5.3e-21), macrophages (aFETp = 4.1e-18), monocytes (aFETp = 5.6e-16), and fibroblasts (aFETp = 1.0e-9) (Fig. 3c). To confirm the cell type predictions from the aggregated network, we further analyzed the cell type composition for each SnG-enriched module. After excluding the modules enriched for the inhibitory gene cluster sc3, we found 62 SnG-enriched modules were significantly enriched for 34 cell-type marker gene signatures (aFETp < 0.01, Fig. 3d, Table S8). The most frequently enriched cell types were fibroblasts (*n* = 44), endothelial cells (*n* = 34), monocytes (*n* = 20), macrophages (*n* = 17), and microglia (*n* = 16), suggesting these cell types were closely associated with SnG expression and regulation. Interestingly, many modules were enriched for multiple cell types. For example, the module M5 from the brain hippocampus, which was most enriched for the CellAge SnGs, significantly overlapped with the markers of the endothelial cells, fibroblasts, and immune cells such as microglia (Fig. 3d). The network modules from different brain regions showed similar cell-type patterns. This co-enrichment of diverse cell types indicated their potential interactions in the senescence response.

To exclude the potential influence of aging on the cell-type enrichment of SnGs, we constructed another set of co-expression networks for 50 tissues by including age as a covariate for adjustment. In the age-adjusted co-expression networks, we identified 107 SnG-enriched modules and 1,779 genes from the aggregated network. In total, 1,619 (91%) of the aggregated network genes were shared between age-adjusted and unadjusted datasets (Fig. S1a). The most frequent SnGs, the cell-type enrichment signatures, and the conservation pattern with other SnG annotation databases were also conserved between the two datasets (Fig. S1b-c). These observations suggest that co-expression patterns of SnGs are robust for the human tissues regardless of age adjustment. Although age had a minor influence on network structures, it still correlated with some gene expressions from the aggregated network. For example, 134 (6.7%) genes showed an increased expression with age in at least five tissues (*p* < 0.05, Spearman correlation, Table S9). Interestingly, *CDKN1A* (*p21*) expression positively correlated with age in 12 different tissues, ranking as the top gene with age correlations. Other well-known senescence genes, such as *ZFP36* and *CYR61*, also had expressions positively correlated with age in seven and five different tissues, respectively. The increased expressions of these SnGs suggest their potential roles in the aging process.

### The SnG co-expression network and cell-type signatures in the brain

**Fig. 4:**
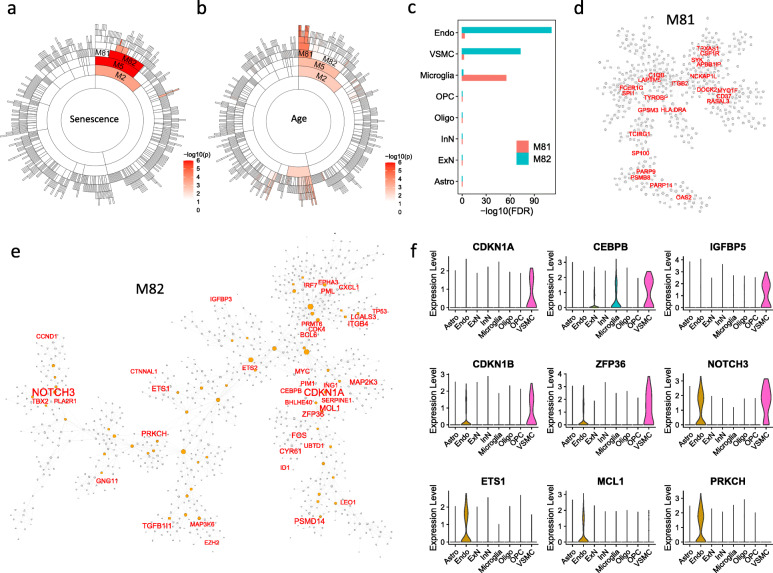
SnG-enriched modules and associated cell types in the brain. **a** Sunburst plot of the SnG-enriched modules in the hippocampus. Each cell showed a hierarchical module from the co-expression network. The color intensity of each cell is proportional to the corresponding enrichment of the CellAge SnGs (FET *p*-value). **b** Sunburst plot of module correlation with donor age. For each network module, the Spearman correlation was calculated between the module eigengene and the donor age. The color intensity of each cell is proportional to the corresponding correlation *p*-value. **c** The enrichment of cell-type marker genes for the modules M81 and M82. For each cell type, the marker genes were identified from the snRNA-seq dataset by the Wilcoxon Rank Sum test. FDR was calculated as the adjusted FET p-value, which tested marker gene enrichment for M81 and the M82. **d** The co-expression network of M81. The hub genes in the module are labeled and shaded with red color. **e** The co-expression network of M82. The CellAge SnGs are labeled and highlighted with red color. The orange nodes are the hub genes. The node sizes are proportional to the connectivity in the network. **f** Violin plot of the SnGs preferentially expressed in VSMCs (top two rows) and endothelial cells (bottom two rows) in the brain snRNA-seq dataset. The x-axis shows the cell types in the scRNA-seq, and the y-axis shows the log-transformed normalized gene expressions

As the bulk RNA-seq networks showed strong senescence signals in different brain regions (Fig. 1b), we further integrated bulk RNA-seq and scRNA-seq data to characterize SnG-enriched modules and cell-type specificity of SnGs in the brain. Among 125 SnG-enriched modules, module M5 from the hippocampus had the highest enrichment value (aFETp = 1.9e-05) of the CellAge SnGs. M5 contained 1,485 genes and could be classified into two sub-modules: M81 with 507 genes and M82 with 976 genes (Table S10). Interestingly, the modules M81 and M82 showed different features. The genes from module M82 were significantly enriched for the CellAge SnGs (aFETp = 2.2e-05, Fig. 4a), while the eigengene of M81 was significantly correlated with age (*p* = 9.1e-05, Spearman correlation, Fig. 4b). To accurately estimate the cell type composition of these two modules, we compared the module genes with the cell-type marker genes from the brain single-nuclei RNA-seq (snRNA-seq) dataset [36]. The snRNA-seq analysis identified 959, 547, and 199 marker genes preferentially expressed in endothelial cells, vascular smooth muscle cells (VSMCs), and microglia, respectively. Among them, 220 endothelial cell marker genes and 137 VSMC maker genes significantly overlapped M82 with FET *p* = 9.0e-113 (FE = 6.1) and 9.3e-74 (FE = 6.6), respectively (Fig. 4c). In contrast, the microglia cell marker genes significantly overlapped M81 (FET *p* = 2.1e-56). Consistently, the co-expression networks from bulk RNA-seq showed that M81 contained several microglia marker genes (e.g., *APBB1IP*, *TYROBP*, and *CSF1R*) as the hub genes, and M82 contained several well-characterized SnGs (e.g., *CDKN1A*, *CEBPB*, *SERPINE1*, and *TP53*) (Fig. 4d-e). To further illustrate cell-type expression profiles of SnGs, we compared the CellAge SnGs with the marker genes of different brain cell types. We found that the CellAge SnGs were significantly enriched for the marker genes of endothelial cells (aFETp = 4.8e-04) and VSMCs (aFETp = 1.1e-08). For example, 24 CellAge SnGs, including *ETS1*, *MCL1*, and *PRKCH*, were marker genes preferentially expressed in endothelial cells, and 25 SnGs (e.g., *CDKN1A*, *CEBPB*, and *IGFBP5*) were preferentially expressed in VSMCs (Fig. 4f). The endothelial cells and VSMCs also shared 13 CellAge SnGs (e.g., *CDKN1B*, *ZFP36*, and *NOTCH3*) as marker genes.

We estimated the proportion of cells enriched for SnGs using the brain snRNA-seq dataset. Similar to previous publications [44, 45], we applied the GSEA method to identify the cells with top-ranked expressions of SnGs by comparing them with permutation trials. The 153 SnGs with inducer functions from the CellAge database were used for GSEA analysis to exclude the genes with potentially unrelated functions. Among the 17,093 single cells profiled in brain tissues, GSEA identified 192 (1.1%) cells significantly enriched for SnGs (*p* < 0.01, 1000 permutations), including 109 endothelial cells, 20 astrocytes, 19 VSMCs, 19 excitatory neurons, 8 microglia cells, 8 oligodendrocyte precursor cells, 6 inhibitory neurons, and 3 oligodendrocytes. For different cell types, the highest fraction of SnG-enriched cells were observed in VSMCs (55.9%), endothelial cells (23.5%), and microglia (6.2%).

We further validated the co-expressions between the aggregated network genes with senescence makers in single cells of the brain. As the dropout effect frequently influences gene expressions in single cells, we performed the coexistence analysis that measured the percentage of cells expressing two target genes simultaneously. Two widely accepted genes, *CDKN1A* (*p21*) and *GLB1* (encoding human senescence-associated β-galactosidase [67]), were chosen as senescence markers. *CDKN1A* and *GLB1* co-expressed in 5.9% VSMCs, 3.1% microglia, and 1.3% endothelial cells, and they were also detected in other cell types including excitatory neurons (0.56%), inhibitory neurons (0.27%), and oligodendrocyte precursor cells (0.12%) (Table S11). The median pairwise coexistence proportions between *CDKN1A* and 77 CellAge-annotated genes from the aggregated network were 2.94, 0.77, and 0.65% in VSMCs, microglia, and endothelial cells, respectively, comparable to the coexistence proportions between *CDKN1A* and the 1924 unannotated genes (2.94, 0.77, and 0.43%, respectively). In contrast, the median coexistence proportions between *CDKN1A* and 1,000 random background genes were 0, 0.77, and 0.22% in the three cell types. Wilcoxon test showed that the coexistence proportions between *CDKN1A* and aggregated network genes significantly increased in VSMCs and endothelial cells compared with random genes (*p* < 4.1e-05). Similarly, the coexistence proportions of *GLB1* in endothelial cells increased from 0.65 to 1.3% (*p* = 1.7e-3, Wilcoxon test) and 1.7% (*p* = 1.6e-35, Wilcoxon test) for the CellAge-annotated and unannotated genes, respectively. Therefore, compared with background genes, both annotated and unannotated genes from the aggregated network showed significant co-expressions with senescence markers in VSMCs and endothelial cells of brain tissues.

**Fig. 5:**
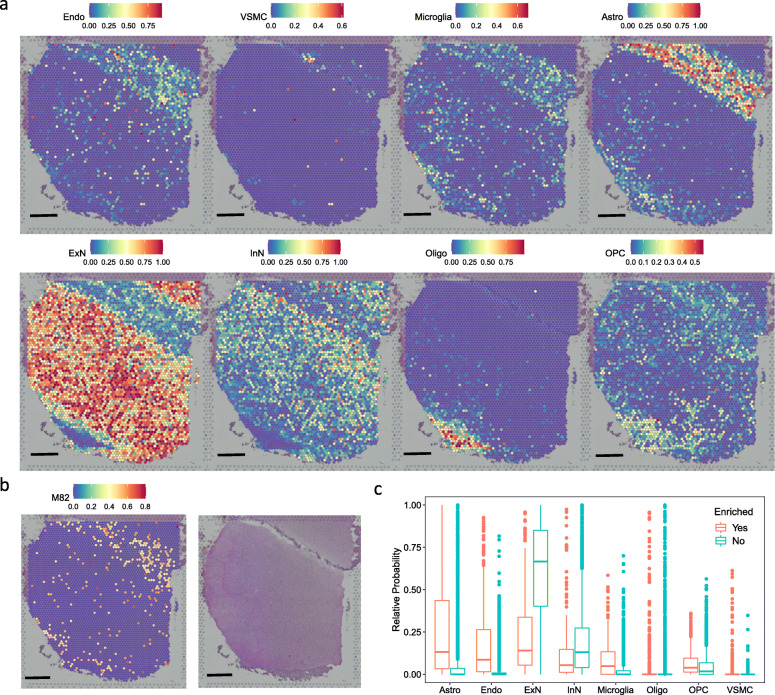
Spatial transcriptomic analysis of M82-enriched cell types in the brain. **a** Cell type inference of spatial voxels from the spatial transcriptomic slide. Gene expressions in the dorsolateral prefrontal cortex were profiled from 10-μm serial tissue sections by the 10x Genomics Visium platform. Color intensity is proportional to cell-type specific gene expression in the snRNA-seq dataset. Scale = 1 mm. **b** The spatial voxels enriched for the module M82 (left) and the corresponding histology slide (right). For each spatial voxel, the enrichment score was calculated by the GSEA method, which tested whether the module M82 genes were enriched in the highly expressed genes. Only spatial voxels with significant enrichment scores (*p* < 0.01, 1,000 permutations) were considered to be enriched for M82. **c** Relative probability of cell type assignment in the M82-enriched voxels versus the voxels that are not enriched for the M82

The cell-type analysis showed that genes in the M82 were enriched for the CellAge SnGs and preferentially expressed in the endothelial cells and the VSMCs. However, such analysis lacked information on cell spatial distribution and co-localization. To further illustrate the spatial localization of M82-enriched cells, we analyzed a spatial transcriptomic dataset of the postmortem human brain using the 10x Genomics Visium platform [42]. By applying an anchor-based integration workflow, we transferred probabilistic cell-type annotations from pre-defined cell types of the scRNA-seq dataset [36]. As each voxel from the spatial transcriptomic slide contained multiple cells from different cell types, we analyzed the probabilistic annotations of the voxels separately for each cell type. Visualization of the spatial transcriptomic slide showed that endothelial cells, microglia, and astrocytes had strong spatial co-localization at the sulcus, while excitatory and inhibitory neurons were organized throughout the cortex (Fig. 5a and b). We further performed GSEA to calculate the enrichment score of the M82 and map M82-enriched voxels [44, 45]. Among all the 4,226 voxels from the spatial transcriptomic slide, 342 voxels (8%) showed significant enrichment (*p* < 0.01, 1000 permutations) of the genes in M82 (Fig. 5b). Spatial localization of the M82-enriched voxels resembled the distribution patterns of endothelial cells, microglia, and astrocytes on the slide. Consistently, compared with the voxels not enriched for the M82, the M82-enriched voxels exhibited higher probabilistic annotations in the cell types including endothelial cells (*p* = 9.6e-116, Wilcoxon test), microglia (*p* = 6.9e-36, Wilcoxon test), and astrocytes (*p* = 6.8e-53, Wilcoxon test), suggesting the M82 genes were expressed in these cell types (Fig. 5c).

### The SnG co-expression network and cell-type signatures in the testis

**Fig. 6:**
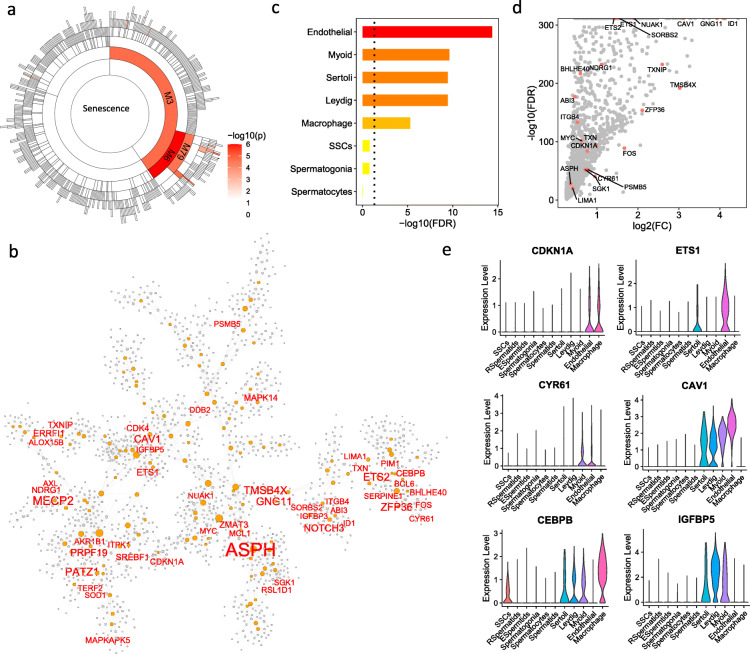
SnG-enriched modules and associated cell types in the testis. **a** Sunburst plot of SnG-enriched modules in the testis. Each cell indicates a network module, and the color intensity indicates the significance of the enrichment for the CellAge SnGs (FET p-value). **b** The co-expression network of module M6. The CellAge SnGs in module M6 are labeled and highlighted with red color. The orange nodes are hub genes, and the node size is proportional to the connectivity in the network. **c** The cell-type specificity of the M6 SnGs. The marker genes for each cell type were identified by the Wilcoxon Rank Sum test from the scRNA-seq dataset. FET was performed to test the enrichment of the marker genes for the CellAge SnGs of the M6. **d** Volcano plot of marker gene expressions in endothelial cells. The x-axis indicates log2 transformed fold change compared with the other cell types. The y-axis indicates the statistical significance from the Wilcoxon Rank Sum test. The CellAge SnGs from M6 are labeled and highlighted in red color. **e** Violin plot of SnG expressions preferentially in endothelial cells (top two rows) and myoid cells (bottom two rows)

According to the gene co-expression networks, the testis was the tissue most enriched for the CellAge SnGs (Fig. 1c). Therefore, we integrated the bulk RNA-seq and scRNA-seq data in the testis to characterize the SnG signatures. In the testis co-expression network, the module M6 contained 1,769 genes and was most enriched for the CellAge SnGs (aFETp = 2.3e-04, Fig. 6a). In M6, 48 (17.2%) genes were annotated by the CellAge database, including well-defined senescence regulators *CDKN1A*, *CEBPB*, *SERPINE1*, *EST2*, *CYR61*, and *MAPK14*/*p38α* (Fig. 6b, Table S12). To investigate the cell types contributing to the CellAge SnGs of M6, we analyzed a scRNA-seq dataset in the testis [38]. We found that the CellAge SnGs from M6 significantly overlapped the marker genes of endothelial cells, macrophages, and the supporting cells including myoid, Sertoli, and Leydig cells (aFETp < 0.05, Fig. 6c). For example, 1,628 marker genes of endothelial cells were identified by the scRNA-seq analysis of the testis (Fig. 6d), and they contained nearly half (*n* = 23, 47.9%) of the CellAge SnGs in the M6 (FE = 8, FET *p* = 4.7e-16) including *CDKN1A* and *EST1* (Fig. 6d-e). Myoid cells are a type of smooth muscle cell that surroundsg the seminiferous tubules. The marker genes of myoid cells were also significantly enriched for the CellAge SnGs of M6 (*n* = 19, FET *p* = 5.2e-11), including *CEBPB* and *IGFBP5* (Fig. 6e). Ten genes (e.g., *CYR61* and *CAV1*) were shared as the marker genes by the endothelial and myoid cells (Fig. 6e). We further performed the GSEA analysis to estimate the proportion of cells enriched for SnGs in the testis. Among the total 6,490 cells, 417 (6.4%) were significantly enriched for the senescence inducers from the CellAge database (*p* < 0.01, 1,000 permutations), including 118 endothelial cells, 107 elongated spermatids, 85 spermatids, 33 Leydig cells, 31 myoid cells, and 25 macrophages. The cell types with the highest percentage of SnG-enriched cells came from endothelial cells (33.4%), myoid cells (10.2%), and Sertoli cells (9.3%).

In the testis tissue, co-expression of *CDKN1A* and *GLB1* was most detected in macrophages (7.3%), endothelial cells (4.0%), and myoid cells (2.0%) (Table S13). The median coexistence proportions between *CDKN1A* and the CellAge-annotated genes from the aggregated network were 2.6%, 4.0%, and 1.0% in macrophages, endothelial cells and myoid cells, respectively, comparable to the coexistence proportions with the unannotated genes. In contrast, the coexistence proportions between *CDKN1A* and 1,000 random background genes were only 0.29%, 0.28%, and 0% in macrophages, endothelial cells and myoid cells, respectively. *GLB1* co-exists with aggregated network genes in more than 1.7% macrophages, 1.6% endothelial cells, and 1.3% myoid cells. Meanwhile, the coexistence proportions between *GLB1* and random background genes were 0.3, 0.3, and 0% macrophages, endothelial cells and myoid cells, respectively. Wilcoxon test showed that both annotated and unannotated genes were significantly co-expressed with *CDKN1A* and *GLB1* greater than the background genes (*p* < 8.4e-05).

### The SnG-enriched cell types in other tissues

**Fig. 7:**
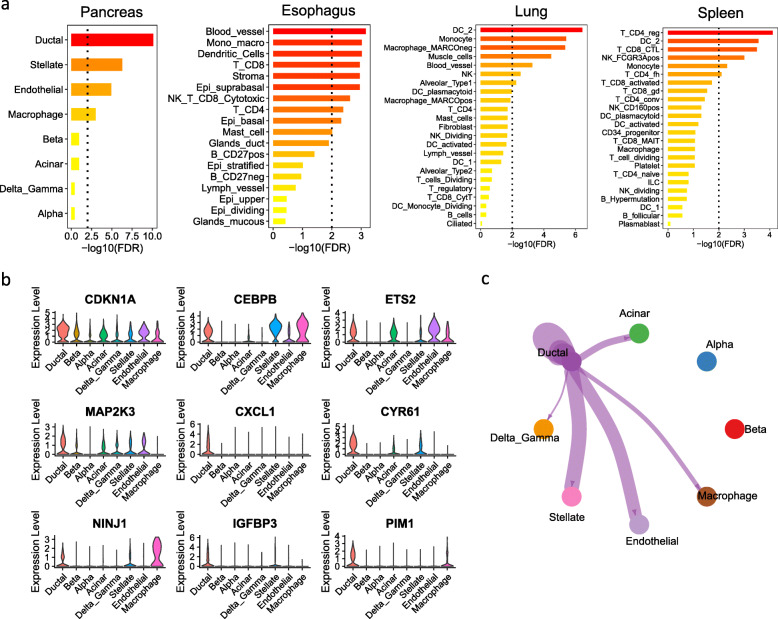
The single-cell analysis of SnG signatures in four different tissues. **a** Enrichment of the 51 conserved SnGs in the cell-type specific marker gene signatures in four tissues. For each tissue, the y-axis shows the cell types identified from the scRNA-seq data, and the x-axis represents the enrichment significance. The dotted line indicates the significance cutoff (aFET*p* = 0.01). **b** The violin plot showing SnGs that are preferentially expressed in the ductal cells of the pancreas. **c** The cell communication network between the ductal cells and other cell types in the pancreas. Cell communication was calculated by the observed ligand-receptor pairs between the sender (ductal) cell type and the receiver cell types. The links in the network plot indicate the significant ligand-receptor interactions (*p* < 0.05, 10,000 permutations) between any two cell types. The line thickness is proportional to the number of ligand-receptor pairs

Apart from the brain and the testis, the SnG-enriched modules were also identified in other tissues such as the pancreas, the esophagus, the lung, and the spleen (Fig. 1b). Therefore, we performed an in-depth analysis of the scRNA-seq data in these four tissues. Using the 51 conserved SnGs from the clusters sc4 (*n* = 11) and sc2 (*n* = 40), we mapped cell types enriched for the conserved SnGs in different scRNA-seq datasets (Fig. 7a). In the pancreas, the ductal cells showed the highest enrichment (aFETp = 7.9e-11), followed by stellate cells (aFETp = 5.1e-07) and endothelial cells (aFETp = 1.2e-05). Among the 51 conserved SnGs, 19 (37%) genes were marker genes of ductal cells (FE = 6.7, FET *p* = 8.7e-12). Several well-defined senescence and SASP regulators, such as *CDKN1A*, *CEBPB*, *EST2*, *MAP2K3*, *CXCL1*, and *CYR61,* were preferentially expressed in ductal cells (Fig. 7b). The marker genes of ductal cells also contained two senescence regulators dependent on the TP53 pathway: the homophilic adhesion molecule *NINJ1* [68] and the *PIM1* protein kinase [69]. These observations suggested that these SnGs were preferentially expressed and co-regulated in the ductal cells. Apart from pancreas tissue, the conserved SnGs were weakly enriched in several cell types of the esophagus, including the blood vessels, stromal cells, and immune cells (Fig. 7a). In the lung and spleen, the enrichment of the conserved SnGs was observed in immune cells, such as dendritic cells, monocytes, macrophages, and T cells. The muscle cells and blood vessels in the lung were also enriched for the conserved SnGs.

As ductal cells in the pancreas showed the most significant enrichment of the conserved SnGs, we further performed cell communication analysis to investigate how ductal cells interact with other cell types. Based on the ligand-receptor pairs from the CellChat database [41], we identified the cell type pairs that showed significantly higher ligand-receptor interactions than background signals (*p* < 0.05, 10,000 permutations). We detected active communications between ductal cells and other cell types in the pancreas, except alpha and beta cells (Fig. 7c, Table S14). The ductal cells secreted 15, 18, 8, and 9 ligand molecules, which formed 43, 37, 19, and 12 ligand-receptor pairs with the receptor genes of stellate, endothelial, acinar, and macrophage cells, respectively. For example, the ductal cells preferentially expressed IL6 class cytokine LIF, which formed ligand-receptor pairs with IL6ST receptor in stellate and endothelial cells. The C3 and JAG, which are ligand proteins in the complement system and NOTCH signaling pathway, were preferentially expressed in ductal cells and were predicted to bind to ITGB2 and NOTCH1 in macrophages. These observations suggested active communications between the SnG-enriched ductal cells and other cell types.

## Discussion

This study presents a comprehensive landscape of SnG co-expression networks and cell-type signatures in healthy human tissues. To date, several studies have examined cell lines and diseased human tissues for the presence of senescent cells [9, 23–25, 70, 71], but a thorough interrogation of healthy human tissues has not been performed. Through an integrative gene network analysis, we identified 125 SnG-enriched co-expression modules spanning 32 different tissues. The gene-module clustering analysis showed that 11 genes from the cluster sc4 and 40 genes from the cluster sc2 were extensively co-expressed in different tissues (Fig. 2a). Consistent with the fundamental roles in regulating cellular senescence, the biological functions of these SnGs are related to regulations of various aspects of cellular senescence, including cell cycle (*CDKN1A* and *TP53*), SASP (*CEBPB*, *MAP2K3, IRF5,* and *IRF7*), mRNA stability (*ZFP36*), wound healing (*CYR61*), apoptosis repression (*MCL1*), enhancers and epigenetics (*FOS* and *ING1a*). In the aggregated network, *CDKN1A* from cluster sc4 was one of the top senescence regulators (Fig. 3a). It was closely co-expressed with interferon signaling genes *STAT3*, *IL4R*, *IL1R1*, *CCL2*, and *IL6* (Fig. 2c), suggesting its central role in connecting cell cycle and SASP production. As our samples were collected from non-diseased human tissues, it is tempting to speculate that *CDKN1A* (*p21*)-mediated senescence represents physiological, non-pathogenic senescence during healthy tissue maintenance. For example, cell renewal and differentiation are controlled by senescence regulators [72], which are similar to the *CDKN1A*-regulated senescence in embryonic development [73, 74]. Alternatively, our study cannot rule out the possibility that we identified resident senescent cells that eventually become toxic; evidence supporting this interpretation includes the cell types identified (i.e.*,* mitotically competent) and their similarities with replicative senescence in cell lines that develop toxic SASP.

Our study revealed that SnGs are co-expressed in different normal human tissues and cell types. This cell-type specific SnG signature and its gene co-expression structure remained stable with age adjustment (Fig. S1), indicating our network biology analysis captures some intrinsic features of SnGs. Meanwhile, age can also influence SnG expressions as many module eigengenes and aggregated network genes showed increased expressions with age. Since fibroblast cell lines have been widely used to investigate SnG functions, the genes in the CellAge database may be biased toward fibroblast signatures and could have contributed to our identification of fibroblasts as a prominent senescent cell type across tissues. However, we also identified endothelial cells and some immune cells (e.g., monocytes, macrophages, and microglia cells) in SnG-enriched modules, suggesting the results captured a senescence signature across multiple mitotically competent cell types in human tissues. Moreover, the senescent cell types we identified have been associated with human diseases in other studies, such as fibroblasts in idiopathic pulmonary fibrosis [8] and endothelial cells in atherosclerosis [75]. We found that endothelial cells, myoid, and other supporting cells in the testis were enriched for SnGs (Fig. 6). Consistently, a previous study showed that peritubular myoid cells underwent striking morphological changes, enhanced β-galactosidase activity, altered nuclear morphology and cellular protein levels in cell culture [76]. In the pancreas, our analysis revealed that senescence marker genes *CDKN1A*, *CEBPB*, *ETS2*, *MAP2K3*, and *CXCL1* were preferentially expressed in ductal cells (Fig. 7). This was also consistent with the observation that primary pancreatic duct epithelial cells showed premature senescence in cell culture, including enlarged flattened morphology and activated β-galactosidase activity [77]. These experimental studies, together with our network and single-cell predictions, suggest certain cell types in healthy human tissues display senescence signatures.

Our findings were also consistent with some, but not all, experimental results previously reported in the literature for disease conditions. For example, we found that endothelial cells and VSMCs, the essential components of the blood-brain barrier and neurovascular unit in the brain, preferentially expressed SnGs (Fig. 4). An increased ratio of senescent endothelial cells was previously observed with advanced aging in the mouse cerebral microcirculation [45]. The senescence- and leukocyte adhesion-associated genes, including *CXCL8* (*IL8*), *SERPINE1* (*PAI-1*), *CXCL1*, *CXCL2*, *ICAM-2*, and *TIE1*, were previously reported as upregulated in the cortical microvasculature with advanced Braak stages [78], supporting the link between tau protein pathogenesis and increased cellular senescence [70, 79]. Our network results also indicate coordinated activities between aging-related immune surveillance and cellular senescence. For example, module M5 in the hippocampus was composed of two sub-modules, the aging-correlated module M81, and the SnG-enriched module M82 (Fig. 4a-b). The modules M81 and M82 were significantly enriched for marker genes from microglia and endothelial cells, respectively (Fig. 4c). Consistently, strong co-localization signals were observed among endothelial cells, microglia, and astrocytes in the spatial transcriptomic analysis (Fig. 5a). It is plausible that senescent endothelial cells and immune surveillance are balanced in healthy conditions, but disruption of such balance in aging or stress stages will drive disease pathology. Studies evaluating senescent cells in human neurodegenerative diseases have identified additional senescent cell types, including neurons [70], astrocytes [80], and oligodendrocyte precursor cells [12]. Given that our analyses were performed in healthy tissues, endothelial cells and VSMCs may become senescent early in disease stages and drive cellular dysfunction of other cell types. However, we cannot rule out the possibility that the SnGs expressed by endothelial cells and VSMCs are physiological and non-pathogenic. Future studies are needed to determine whether endothelial cells expressing the SnG profile have altered function, pathogenic morphology, or deleterious SASP secretion.

As the network analysis was based on the curated SnGs in the CellAge database, our results may only capture a subset of senescence signatures, primarily driven by previously established knowledge. Cellular senescence triggered by clinical pathologies, which was not experimentally tested in vitro or difficult to culture cell lines (e.g.*,* neurons and oligodendrocytes in the brain), would be missed. Consistently, although our analysis identified *TP53* and *CDKN1A* (*p21*)-centered regulators, the conserved SnGs did not include the other senescence regulators such as *CDKN2A* (*p16*) and *RB1*. This result may reflect different aspects of cellular senescence controlled by the two gene pathways. As in vivo expression levels and cell-type specificity of these two genes have not been elucidated in healthy human tissues, experiments comparing senescence phenotypes between healthy and diseased human tissues are needed to verify this finding. Nevertheless, our results identified novel senescence-related genes not yet experimentally validated, which can be used to generate novel hypotheses regarding cellular senescence. Specifically, we identified 2001 genes in the aggregated network co-expressed in at least five tissues. Among them, only 77 (4%) genes were annotated by the CellAge database, and 345 (17%) genes overlapped replicative signatures identified by meta-analysis [65]. Therefore, the majority of network genes have not been functionally validated. Similar to annotated genes, the unannotated genes from the aggregated network were also co-expressed with senescence markers in single cells of different tissues (Tables S11 and S13). Some of the network genes encode SASP factors induced in senescent cells and regulated by *CDKN1A*, suggesting their potential functions in cellular senescence (Table S7). These novel genes are top candidates for future functional experimental studies to investigate their roles in senescence.

In scRNA-seq experiments, gene dropout effects and the biased cell types from cell dissections [81] may inhibit the efficiency of capturing the full senescence signature across all cell types. Also, our cell-type analysis was based on the relative expression of marker genes across different cell types. It may miss senescence signals when only a small fraction of cell populations are affected (e.g., neuron cells in the brain). Future studies using transcriptomics, both dissociative and spatially revolved platforms, from tissues across the lifespan and in different disease states are needed to better understand the role of senescent cells in health and disease.

## Conclusions

Our study provides a comprehensive landscape of SnG co-expression signatures and their cell-type specificity in 50 healthy human tissues. We identified SnG-enriched gene modules, characterized SnG co-expression patterns, and constructed aggregated SnG networks across primary tissues of the human body. Our network approaches identified 51 conserved SnGs in different human tissues. Further analyses of snRNA-seq and spatial transcriptomic data validated the cell-type specificity of SnG-enriched modules. The landscape of the co-regulated organizations and cell-type specificity of SnGs can serve as a blueprint for future studies to map senescent cells and their cellular interactions in human tissues.

## Supplementary Information

**Additional file 1: Figure S1.** The cell-type enrichment of aggregated SnG network with age adjustment.

**Additional file 2: Table S1.** The SnG-enriched modules in 50 human tissues. **Table S2.** The k-means clusters of SnGs. **Table S3.** The k-means clusters of SnG-enriched modules. **Table S4.** The conserved 3-layer neighborhood of *CDKN1A* in the co-expression networks. **Table S5.** The conserved 3-layer neighborhood of *TP53* in the co-expression networks. **Table S6.** The aggregated network of SnG-enriched modules. **Table S7.** The SASP factors from the aggregated network and their regulations by senescence and *CDKN1A*. **Table S8.** The cell types enriched (FDR < 0.01) for each SnG-enriched module. **Table S9.** The age correlation of aggregated network genes. **Table S10.** The modules M81 and M82 in the brain hippocampus. **Table S11.** Gene coexistence proportion between senescence markers and aggregated network genes in single brain cells. **Table S12.** The module M6 in the testis. **Table S13.** Gene coexistence proportion between senescence markers and aggregated network genes in single testis cells. **Table S14.** The Cell communications between pancreas ductal cells and other cell types.

## Data Availability

The MEGENA co-expression networks and gene modules for 50 tissues are available through GitHub (https://github.com/penguab/GTEx-Networks-For-Senescence). The age-adjusted networks are shared in https://github.com/penguab/GTEx-Networks.

## Abbreviations

SnGs: senescence genes
GTEx: Genotype-Tissue Expression Project
SASP: senescence-associated secretory phenotype
scRNA-seq: single-cell RNA-seq
KNN: K-nearest neighbor
MEGENA: Multiscale Embedded Gene Co-expression Network Analysis
FET: Fisher’s exact test
FDR: False Discovery Rate
GSEA: Gene set enrichment analysis
FE: fold enrichment
aFETp: adjusted FET *p*-value
snRNA-seq: single-nuclei RNA-seq
VMSCs: vascular muscle cells
