# Supplementary material for: The landscape of human tissue and cell type specific expression and co-regulation of senescence genes
Source: Mol Neurodegener. 2022 Jan 9;17:5. doi: 10.1186/s13024-021-00507-7 (PMC8744330; doi:10.1186/s13024-021-00507-7)
Supplement: Supplementary file 1 — Additional file 1: Figure S1. The cell-type enrichment of aggregated SnG network with age adjustment. [file 13024_2021_507_MOESM1_ESM.docx]

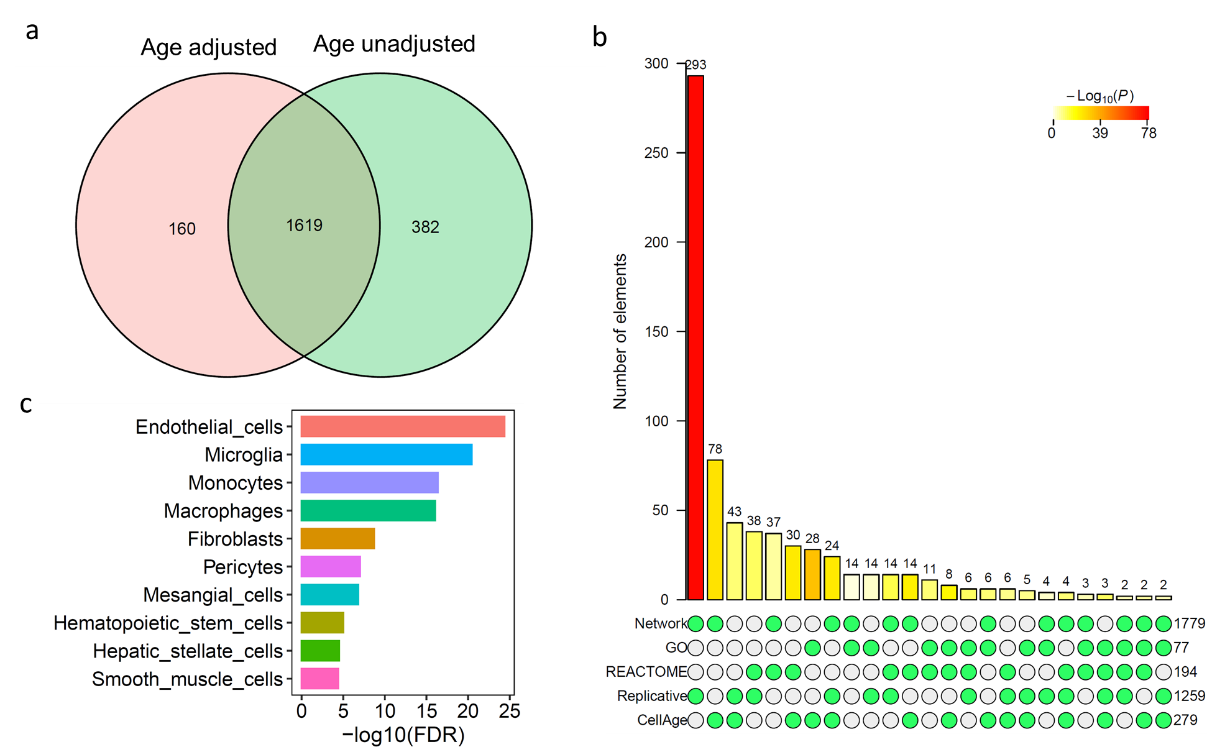


**Fig. S1.** **The cell-type enrichment of aggregated SnG network with age adjustment.** **a)** Aggregated network genes with age-adjusted and unadjusted datasets. **b)** Comparison of the age-adjusted aggregated network with four SnG annotation datasets. **c)** Barplot showing the top 10 cell types enriched (FRD < 0.05) for the age-adjusted aggregated network genes.
